# Resolution enhancement in neural networks with dynamical synapses

**DOI:** 10.3389/fncom.2013.00073

**Published:** 2013-06-11

**Authors:** C. C. Alan Fung, He Wang, Kin Lam, K. Y. Michael Wong, Si Wu

**Affiliations:** ^1^Department of Physics, The Hong Kong University of Science and TechnologyHong Kong, China; ^2^State Key Laboratory of Cognitive Neuroscience and Learning, School of Cognitive Neuroscience, Lab of Neural Information Processing, Beijing Normal UniversityBeijing, China

**Keywords:** continuous attractor neural network, neural field model, short-term synaptic depression, short-term synaptic plasticity, transparent motion

## Abstract

Conventionally, information is represented by spike rates in the neural system. Here, we consider the ability of temporally modulated activities in neuronal networks to carry information extra to spike rates. These temporal modulations, commonly known as population spikes, are due to the presence of synaptic depression in a neuronal network model. We discuss its relevance to an experiment on transparent motions in macaque monkeys by Treue et al. in 2000. They found that if the moving directions of objects are too close, the firing rate profile will be very similar to that with one direction. As the difference in the moving directions of objects is large enough, the neuronal system would respond in such a way that the network enhances the resolution in the moving directions of the objects. In this paper, we propose that this behavior can be reproduced by neural networks with dynamical synapses when there are multiple external inputs. We will demonstrate how resolution enhancement can be achieved, and discuss the conditions under which temporally modulated activities are able to enhance information processing performances in general.

## 1. Introduction

An important issue in computational neuroscience is how information is represented in the neural system. It was widely accepted that spike rates of neurons carry information. This notion was further illustrated in *population codes*, in which the a group of neurons encode information and even represent uncertainties therein through their collective activities (Zemel and Dayan, 1999; Pouget et al., 2000). Consequently, population coding has been successfully applied to describe the encoding of spatial and directional information, such as orientation (Ben-Yishai et al., 1995), head direction (Zhang, 1996), and spatial location (Samsonovich and McNaughton, 1997). They are also used to explain information processing in the recently discovered grid cells (Fuhs and Touretzky, 2006).

An interesting question arises, namely, whether information can be encoded in other aspects of population coding besides spike rates. For example, can extra information be carried by the coding if the spikes are modulated in time, so that different spike trains modulated differently may convey different messages even though their spike rates appear to be the same. Given this possibility, the information content of population coding can be much richer than its superficial appearance as spike rates.

In this paper, we will explore the ability of population spikes to carry information extra to spike rates. Population spikes are temporal modulations of the population neuronal activity, and are also known as ensemble synchronizations, representing extensively coordinated rises and falls in the discharge of many neurons (Loebel and Tsodyks, 2002; Holcman and Tsodyks, 2006). The population spikes are due to the presence of short-term depression (STD) of the synapses, referring to the reduction of synaptic efficacy of a neuron after firing due to the depletion of neurotransmitters (Stevens and Wang, 1995; Markram and Tsodyks, 1996; Dayan and Abbott, 2001). This adds to a recently expanding list of the roles played by STD in neural information processing. For example, STD was recently suggested to be useful in expanding the dynamic range of the system (Abbott et al., 1997; Tsodyks and Markram, 1997), estimating the information of the pre-synaptic membrane potential (Pfister et al., 2010), and stabilizing the self-organized critical behavior for optimal computational capabilities (Levine et al., 2007). STD was also found to be useful in enhancing the mobility of the network state in tracking moving stimuli (Fung et al., 2012a), and hence was recently proposed to be a foundation of a potential anticipation mechanism (Fung et al., 2012b).

Previously, population spikes were found to be global synchronizations of neuronal activities. However, in order for them to encode spatial information, the population spikes that will be considered in this paper are localized ones. We will use the case of transparent motion as an example. This example illustrates the possibility that the modulation by population spikes enables the neural system to refine the resolution of direction for multiple stimuli. The prediction by the proposed mechanism has an excellent agreement with experimental results (Treue et al., 2000).

Transparent motion is one of the most well-known experiments in the psychophysical community. In the experiment, the stimulus usually contains moving dots with different directions. So, there are multiple moving directions transparently superimposed on one another. In the nervous system, the middle temporal (MT) area was found to be responsible for detecting moving directions of objects (Maunsell and Essen Van, 1983). Here, it was recently found that the neurons are heterogeneous, with some neurons responding to the pattern of moving stimuli, while others responding to the components of composite moving patterns (Rust et al., 2006). In 2000, Treue et al. found that if the directions of two groups of moving dots differ by an angle larger than the tuning width of the neurons, the observed neuronal response profile begins to split (Treue et al., 2000). However, subjects can still distinguish the two directions if their difference is as small as about 10° (Mather and Moulden, 1980), while the average direction tuning width of neurons is about 96°.

To resolve this paradox, Treue et al. proposed that when the resultant neuronal response is too board for a single direction, the perception can identify the two directions by considering the resultant neuronal response as a superposition of two individual neuronal responses of each direction. However, when the two directions differ by an angle less than the tuning width, it becomes difficult to resolve the peaks of the two superposed responses, if the curvature of the average neural activity profile is not taken into account This difficulty was also observed in simulations with distributional population codes (Zemel and Dayan, 1999). The mechanism of enhanced resolution remained unknown, and coding by firing rates may not reveal the complete picture.

In a recently proposed model on motion transparency, the enhanced resolution was achieved (Raudies et al., 2011). Two mechanisms held the key to this advance. First, as in standard neural field models, there is a local center-surround competition in the space of motion directions. Although this is not sufficient to explain the enhanced resolution, there is the second mechanism, namely, the modulatory feedback signals from higher stages of processing in the area medial superior temporal (MST) area. Motion attraction (that is, under-estimation of the directional difference) at small angular difference, and motion repulsion (that is, over-estimation) at larger angles were successfully explained. Perception repulsion can also be found in a Bayesian inference explanation on identification of audiovisual stimulus (Sato and Toyoizumi, 2007).

Here, we propose a novel mechanism for resolution enhancement based on the temporal modulation inherent in population coding. To focus on the generic issue of whether information carried in the temporal modulation of population coding can be usefully applied in a processing task, we consider a simplified model of transparent motion. We assume that inputs from different locations of the receptive field have been integrated, the directional information has been filtered, and the processing of input information can proceed without the assistance of feedback modulations. Thus our working model reduces to a single network. The working principle is a continuous attractor neural network (CANN) with dynamical synapses. Continuous attractor neural networks, also known as neural field models, are models used for describing phenomena and features observed in some brain regions where localized attractor neuronal responses are used to represent continuous information. Due to short-range excitatory interactions and long-range/global inhibitory interactions, bump-shaped neuronal response profiles are attractors of CANNs. Since the response profiles are easy to shift their positions in the space of continuous information, they are useful in tracking moving stimuli (Amari, 1977; Ben-Yishai et al., 1995; Wu et al., 2008; Fung et al., 2010) and their drifting behaviors have been studied (Itskov et al., 2011). In contrast to these studies of tracking, we will focus on stationary stimuli and their time-dependent neuronal responses.

Dynamical synapses are found to enrich the dynamical behaviors of CANNs (York and van Rossum, 2009; Fung et al., 2012a). Short-term synaptic depression (STD) can degrade the synaptic efficacies between neurons, depending temporally on the activity history of the presynaptic neuron (Tsodyks et al., 1998). In the presence of an external stimulus, the bumps can remain temporally stable if STD were absent. However, with STD, the population activity may drop after it reaches a maximum, since neurotransmitters have been consumed. After the drop, neurotransmitters are recovered and the neuronal population is ready to respond to the external stimulus again. This results in periodic bursts of local neuronal responses, referred to as population spikes. As we shall see, the temporal modulation induced by STD, together with input fluctuations, enable the system to reduce the angle of resolution in transparent motion down to one-fourth to one-third of the tuning width of the neuron.

In the rest of this paper, we will begin with an introduction of the CANN model and its basic properties. After that, we will discuss simulation results showing that our model is able to represent acute difference in transparent stimuli. At the end, there is a discussion section concluding our proposed mechanism.

## 2. Model and method

In the continuous attractor neural network model, we specify the dynamics and the state of the system by the neuronal current. For neurons with preferred stimulus *x* in the range −*L*/2 ≤ *x* ≤ *L*/2, its neuronal current is denoted by *u*(*x*, *t*). The dynamics of *u*(*x*, *t*) is given by Fung et al. (2012a)

(1) $$\tau_{s}\frac{du}{dt}(x,t)=-u(x,t)+I^{\text{ext}}(x,t)+\rho \int dx^{\prime }J(x-x^{\prime })p(x^{\prime },t)r(x^{\prime },t).$$

τ_*s*_ is the timescale of *u*(*x*, *t*). It is usually of the order of the magnitude of 1 ms. ρ is the density of neurons over the space spanned by {*x*}. *J*(*x* − *x*′) is a translational invariant excitatory coupling given by

(2) $$J(x-x^{\prime })=\frac{J_{0}}{\sqrt{2\pi }a}\exp\left(-\frac{{\left|x-x^{\prime }\right|}^{2}}{2a^{2}}\right),$$

where *a* is the range of excitatory connection and *J*_0_ is the average strength of the coupling. *r*(*x*, *t*) is the neural activity related to *u*(*x*, *t*) by

(3) $$r(x,t)=\Theta \left[u(x,t)\right]\frac{u{\left(x,t\right)}^{2}}{B(t)}.$$

Here, Θ is a step function centered at 0. The denominator, *B*(*t*) ≡ 1 + *k*ρ ∫ *dx*′ *u*(*x*′, *t*)^2^, in this formula is the global inhibition, controlled by the inhibition parameter *k*. This type of global inhibition can be achieved by shunting inhibition (Heeger, 1992; Hao et al., 2009). *I*^ext^(*x*, *t*) is the external input to the system, which will be defined in the latter part of this section.

In the integral of Equation (1), *p*(*x*, *t*) is the available fraction of neurotransmitters of the presynaptic neurons. Neurotransmitters are consumed when a neuron sends chemical signals to its postsynaptic neurons. However, the recovery time of the neurotransmitters is considerably longer than τ_*s*_. This process can be modeled by Tsodyks et al. (1998) and Fung et al. (2012a)

(4) $$\tau_{d}\frac{dp}{dt}(x,t)=-p(x,t)+1-\tau_{d}\beta p(x,t)r(x,t).$$

τ_*d*_ is the timescale of recovery process of neurotransmitters. The recovery process usually takes 25–100 ms. Here, we choose τ_*d*_ = 50τ_*s*_. These two differential equations, Equations (1) and (4), are found to be consistent with the model proposed by Tsodyks et al. (1998).

The stimulus fed to the system consists of *n* components, each with a Gaussian profile and a time-dependent fluctuation in strength. It is given by

(5) $$I_{0}^{\text{ext}}(x,t)=\sum_{i=1}^{n}\left[A_{0}+\delta A_{i}(t)\right]\exp\left(-\frac{{\left|x-z_{i}\right|}^{2}}{2a_{I}^{2}}\right).$$

Here, *z*_*i*_'s are the peak positions of the components, and *a*_*I*_ is the width of the Gaussian profiles. If not specified, it was assumed to be the same as the synaptic interaction range *a* used in Equation (2). *A*_0_ is the average relative magnitude of one input component, while δ*A*_*i*_(*t*) is a random fluctuation with standard deviation σ_*A*_ in amplitude of input components.

**Figure 1:**
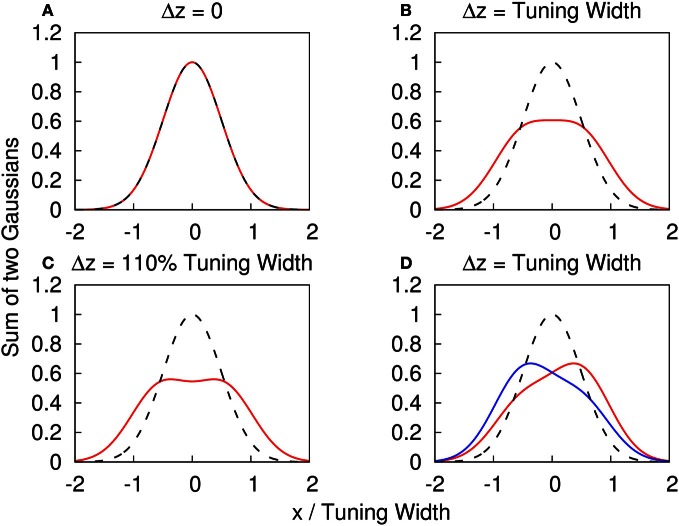
**(A–C)** The profile of two superposed Gaussian functions with the same height. *f*(*x*) ≡ {exp[(*x* − Δ*z*/2)^2^/(2*a*^2^)] + exp[(*x* + Δ*z*/2)^2^/(2*a*^2^)]}/2. Red solid line: *y* = *f*(*x*) with different Δ*z*. Dashed line: *y* = *f*(*x*) with Δ*z* = 0 as a reference. **(A)** Δ*z* = 0. **(B)** Δ*z* = tuning width = 2*a*. **(C)** Δ*z* = 110% tuning width = 2.2*a*. **(D)** The profile of two superposed Gaussian functions with different heights to illustrate how the amplitude fluctuations provide a cue to distinguish the components. *g*(*x*) ≡ {*A*_0_ exp[(*x* − Δ*z*/2)^2^/(2*a*^2^)] + *A*_1_ exp[(*x* + Δ*z*/2)^2^/(2*a*^2^)]}. Dashed line: *y* = *f*(*x*) with Δ*z* = 0 as a reference. Red solid line: *y* = *g*(*x*) with Δ*z* = tuning width, *A*_0_ = 0.4 and *A*_1_ = 0.6. Blue solid line: *y* = *g*(*x*) with Δ*z* = tuning width, *A*_0_ = 0.6 and *A*_1_ = 0.4.

Note that when the Gaussian profiles have strong overlaps, the components cannot be resolved, as illustrated in Figures 1A–C. We consider the amplitude fluctuations of each component to be independent of each other, i.e., 〈δ*A*_*i*_δ*A*_*j*_〉 = 0, where the average is over time. These fluctuations provide a cue for the system to distinguish different components (Figure 1D). This is consistent with the psychophysical experiment which showed that spatial and temporal randomness is important for perception of motion transparency (Qian et al., 1994). Since the fluctuations vanish when averaged over time, a system responding only to time-averaged inputs is unable be able to detect the components. Here, the role of STD is to modulate the network state, so that it responds to one input component once a time.

To model the situation that the maximum strength of the input profile is invariant, we consider the input in Equation (1) to be

(6) $$I^{\text{ext}}(x,t)=\frac{A}{\max_{x}\left[I_{0}^{\text{ext}}(x,t)\right]}I_{0}^{\text{ext}}(x,t),$$

where *A* is the fixed maximum magnitude of the external input. As the external input profile is set to have a constant maximum, only the ratio σ_*A*_/*A*_0_, rather than the magnitudes of *A*_0_ and σ_*A*_, is relevant in our studies.

It is convenient to rescale the dynamical variables as follows. We first consider the case without STD when β = 0, and the synaptic interaction range *a* « *L*. In this case, *p*(*x*, *t*) = 1 in Equation (1). For $k\leq k_{c}\equiv \rho J_{0}^{2}/(8\sqrt{2\pi }a)$, the network holds a continuous family of Gaussian-shaped stationary states when *I*^ext^(*x*, *t*) = 0. These stationary states are

(7) $$\tilde{u}(x)={\tilde{u}}_{0}\exp\left(-\frac{{\left|x-z\right|}^{2}}{4a^{2}}\right)\;,$$

and

(8) $$\tilde{r}(x)={\tilde{r}}_{0}\exp\left(-\frac{{\left|x-z\right|}^{2}}{2a^{2}}\right)\;.$$

where $\tilde{u}(x)$ is the rescaled variable ρ*J*_0_*u*(*x*), and ${\tilde{u}}_{0}$ is the rescaled bump height. The parameter *z*, i.e., the center of the bump, is a free parameter, implying that the stationary state of the network can be located anywhere in the space *x*. In this paper, we assume that the variable is represented solely by the peak position of the neural activity profile. This assumption is one of the most direct ways to interpret the population code. However, there are other ways to interpret population codes. For example, Treue et al. (2000) proposed that the curvature of the average of the neural activity carries information represented by the neural population code, although the mechanism achieving this objective is not clear (Treue et al., 2000). On the phenomenological level, distributional population coding and double distributional population coding were proposed to represent information in population coding with more sophistication (Zemel and Dayan, 2000; Sahani and Dayan, 2003).

The tuning width of a neuron, defined as the standard deviation of the firing rate profile multiplied by 2, is therefore 2*a*. In the present work, we rescale the neuronal current as $\tilde{u}(x,t)\equiv \rho J_{0}u(x,t)$, together with the corresponding rescaling of other variables given by $\tilde{A}\equiv \rho J_{0}A,\;\tilde{k}\equiv k/k_{c},\;\tilde{\beta }\equiv \tau_{d}\beta /\left(\rho^{2}J_{0}^{2}\right)$. By using these rescaling rules, the dynamics of the system should only depend on $\tilde{k}$, $\tilde{\beta }$, τ_*d*_/τ_*s*_, σ_*A*_/*A*_0_, *z*_*i*_'s and $\tilde{A}$. Below, only these parameters will be specified.

In each simulation, the variables *u*(*x*, *t*) are modeled to be located at *N* discrete positions uniformly distributed in the space of preferred stimuli {*x*}. To do massive simulations, all simulation results are generated by using *N* = 80. We have verified that the dynamics of the system is independent to *N*, and the number of neurons should not affect the conclusion. The boundary condition of the space is periodic. The range of the network is 360° and the tuning width of the neurons is 96°, following the experimental estimates in Treue et al. (2000). To solve differential equations in Equations (1) and (4), we used the Runge-Kutta Prince-Dormand (8,9) method provided by the GNU Scientific Library. Initial conditions of *u*(*x*, *t*)'s is zero, while *p*(*x*, *t*)'s are initially 1. The local error of each evolution step is less than 10^−6^. The random number generator used to generate the Gaussian random number is the generator proposed by Lüscher (1994). The Gaussian fluctuation is updated every 50τ_*s*_.

## 3. Results

### 3.1. Population spikes

**Figure 2:**
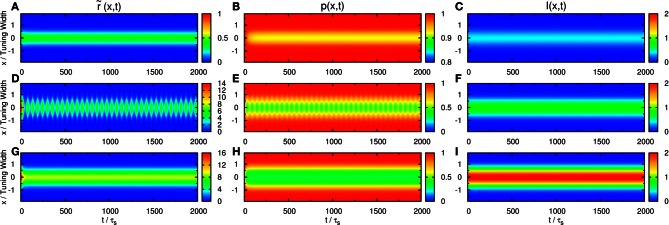
**Firing rates $\tilde{r}(x,\;t)$ (A, D, and G), available fraction of neurotransmitters *p*(*x*, *t*) (B, E, and H) and corresponding input (C, F, and I) for various magnitudes of single-peaked external inputs. (A–C)** $\tilde{A}=0.4$, **(D–F)** $\tilde{A}=0.8$, and **(G–I)** $\tilde{A}=2.0$. Other parameters: $\tilde{k}=0.5$, $\tilde{\beta }=0.24$, *a* = 48π/180, and τ_*d*_ = 50τ_*s*_.

We first consider the response of the network when the input consists of one component. We explore the network behavior by varying the parameters $\tilde{k}$, $\tilde{\beta }$, and $\tilde{A}$. We found a rich spectrum of behaviors including population spikes, static bumps, and moving bumps. The full picture will be reported elsewhere. For the purpose of the present paper, we fix $\tilde{k}$ and $\tilde{\beta }$ at a typical value and consider the behavior when $\tilde{A}$ increases. As shown in the top panel of Figure 2, the network cannot be triggered to have significant activities when the input is weak. In the bottom panel, the input is so strong that the network response is stabilized to a static bump with time-independent amplitude. An interesting case arises in the middle panel for moderately strong input, where population spikes can be observed. Population spikes are the consequence of the presence of STD. They are caused by a rapid rise of neuronal activity due to the external stimulus. Then in a time of the order of τ_*d*_, the neurotransmitters are consumed, leading to a rapid drop in neuronal activity. When the neurotransmitters recover, the neurons become ready for the next population spike, resulting in the interesting periodic behavior. Population spikes have been found before as synchronization of neuronal activities, and their potential role in processing information was appreciated, but no specific context of such applications was identified (Loebel and Tsodyks, 2002), Here, we will present an example that spatially localized population spikes endow the neural system a capacity of reading-out input components.

### 3.2. Network activities for two stimuli

Next, we consider inputs with two components separated by Δ*z* > 0 and study the network behavior when Δ*z* gradually increases. Without loss of generality, we choose *z*_1_ = Δ*z*/2 and *z*_2_ = −Δ*z*/2. The relative fluctuation is σ_*A*_/*A*_0_ = 0.3.

**Figure 3:**
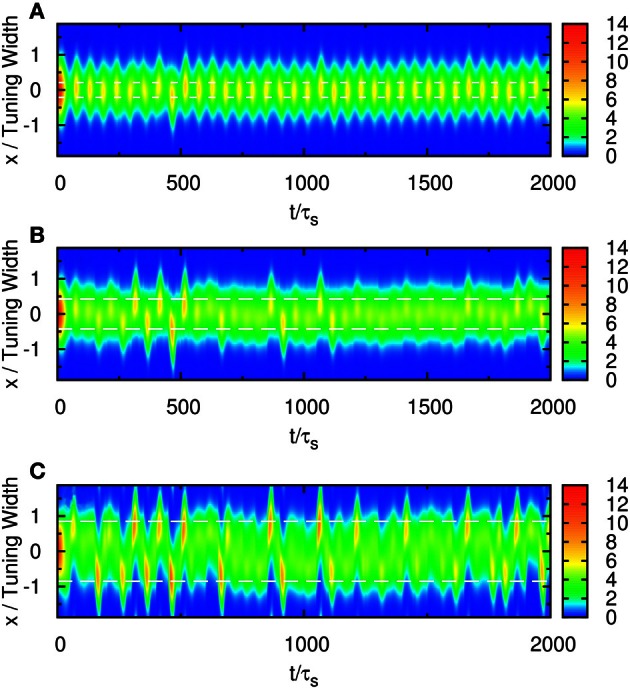
**Raster plot of firing rates $\tilde{r}$ for (A) Δ*z* = 0.5, (B) Δ*z* = 1.0, and (C) Δ*z* = 2.0.** White dashed lines: positions of stimuli. Parameters: other parameters: $\tilde{k}=0.5$, $\tilde{\beta }=0.24$, *a* = 48π/180, $\tilde{A}=0.8$, σ_δ*A*_*i*__/*A*_0_ = 0.3, and τ_*d*_ = 50τ_*s*_.

When the separation is small, the positions of the population spikes fluctuate around the mid-position of the two stimuli, as illustrated in Figure 3A. The two components cannot be resolved.

When the separation increases to the extent that the two components remain barely resolved, an interesting change in the spiking pattern occurs as shown in Figure 3B. The positions of the population spike peaks begin to center around the two input components, although the shoulders of the population spikes remain overlapping considerably. Note that in this regime, the profile of the neuronal activities remain unresolved when they are averaged over time. However, due to the presence of STD, it is likely that a population spike is produced at the position of the component which happens to be higher due to height fluctuations. Hence in this regime, the population spike peaks are no longer aligned at the center. Rather, they are arranged in two rows, each around the two components. Furthermore, the two rows of population spikes tend to fire alternately. This implies that although it is hard to resolve the two components by considering the time-averaged signals, the temporal modulation by the alternating population spikes may be utilized for resolution enhancement.

When the separation increases further, the population spikes form two groups clearly, as shown in Figure 3C. The two components are clearly resolved.

**Figure 4:**
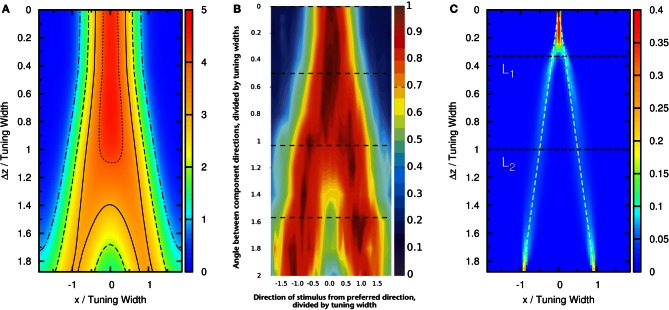
**(A)** Time average of firing rates $\tilde{r}$ as a function of the preferred stimuli of neurons, *x*, and the separation between the two stimuli, Δ*z*. Contour lines: ${\langle \tilde{r}\rangle }_{t}=1$ (dotted-dashed line), ${\langle \tilde{r}\rangle }_{t}=2$ (dashed line), ${\langle \tilde{r}\rangle }_{t}=3$ (solid line), ${\langle \tilde{r}\rangle }_{t}=4$ (dotted line). Parameters: same as Figure 3. **(B)** The average neural activity recorded by Treue et al. (2000) (with license number 3125800919243 for the reuse purpose). **(C)** Contours of the distribution of peak positions higher than 6.2 as a function of preferred stimuli, *x*, and the separation between the two stimuli, Δ*z*. White dashed line: positions of the two stimuli. *L*_1_, one-third of the tuning width. *L*_2_, tuning width. Parameters, same as Figure 3.

To compare our model with experimental results, we measure the time average of neuronal activities as a function of preferred stimuli of neurons and the separation of the two stimuli, shown in Figure 4A. We found that this result is very similar to the experimental results reported by Treue et al. [Figure 2C in Treue et al. (2000)]. The peak of the average profile of neuronal activities splits near Δ*z* ~ 1.0× tuning width. However, the time-averaged data cannot explain why subjects can resolve separations much less than the tuning width.

### 3.3. Extraction of modulated information

**Figure 5:**
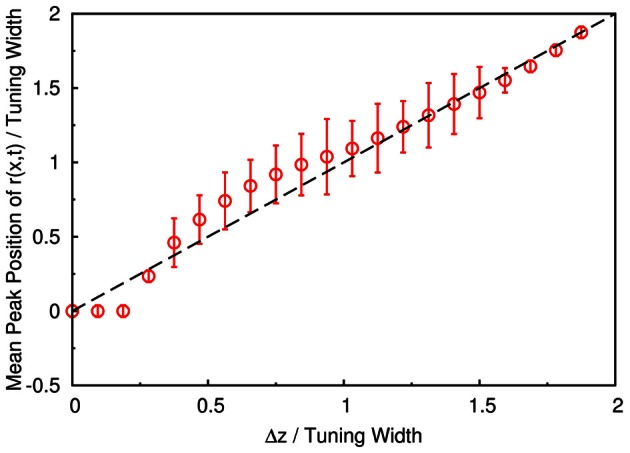
**The mean separation of peak positions of *r*(*x*, *t*) shown in Figure 4C.** Symbols, simulation. Dashed line, diagonal line representing perfect distinguishability.

To demonstrate that the neuronal activities carry the information about two stimuli, we collect statistics on the peak positions of the population spikes. Here the peak position is calculated by $\max_{x}\tilde{r}(x)$. In Figure 4C, we present the contour plot of the distribution of peak positions in the space of the preferred stimuli of neurons and separation between the two stimuli in units of the tuning width. To focus on peaks with significant information only, we counted only population spikes with maximum amplitudes above an appropriately chosen threshold. Each column in Figure 4C is a normalized histogram with 80 bins. In order to obtain a relatively smooth distribution, the sampling process lasted for 100,000 τ_*s*_. The mean of the separation between peak positions is plotted in Figure 5 as a function of Δ*z*. We found that in this setting, the system can detect the input separation down to one-fourth of the tuning width. We note that in Figure 4C, when the difference between the components is too small, Δ*z* ≲ 1/4 tuning width, population spikes occur at the middle of the net external input profile with a relatively small variance. However, when the network starts to resolve the two components, there are notable variances on positions of the population spikes in each component. The standard deviation of the positions of the population spikes in each component is roughly of the order of 0.1 times the tuning width, which is roughly 20°, as shown in Figure 5.

To investigate whether the statistics with long sampling period is applicable to sampling periods in actual experiments, we have also collected statistics for 500τ_*s*_. (In the experiment done by Treue et al., subjects took 500 ms to perform the discriminational task.) The result is shown in Figure A2A in Appendix. Although the distribution is rougher because of the relatively small sampling size, enhanced resolution down to 0.3 tuning width is still visible.

Furthermore, when the separation between the two stimuli lies between one-third and three-halves of the tuning width, the system slightly overestimated the separation of the two profiles. If we take the tuning width to be 96° (Treue et al., 2000), this range will be approximately from 30° to 140°. This is consistent with the experimental results of Braddick et al. (2002), in which subjects overestimated some moving direction difference in transparent motion experiments. However, it was reported in Figure 4 in Treue et al. (2000) that the perceived separation of movement direction starts to underestimate the truth when the stimulus separation increases above 40°. Since the range corresponding to “motion repulsion” reported by Braddick et al. (2002). is different from that reported by Treue et al., it seems that the range of differences between stimuli corresponding to “motion repulsion” is different for different experimental settings.

**Figure A1:**
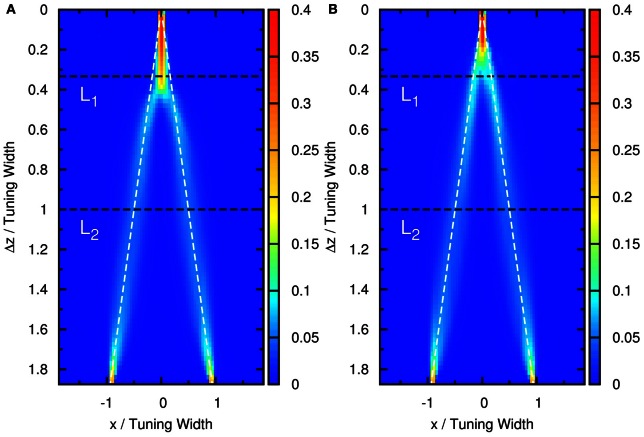
**Contours of the distribution of peak positions higher than (A) 6.2 and (B) 5.5 as a function of preferred stimuli, *x*, and the separation between the two stimuli, Δ*z*.** White dashed line: positions of two stimuli. Parameters: same as Figure 3, except *a*_*I*_ = 0.95*a* for **(A)** and *a*_*I*_ = 1.05*a* for **(B)**.

We have also tested the effects of choosing the widths of external input components to be different from the tuning width of the neuronal response. We found that the results for different stimulus strengths in Figure A1 in Appendix are qualitatively the same as that in Figure 4C.

**Figure 6:**
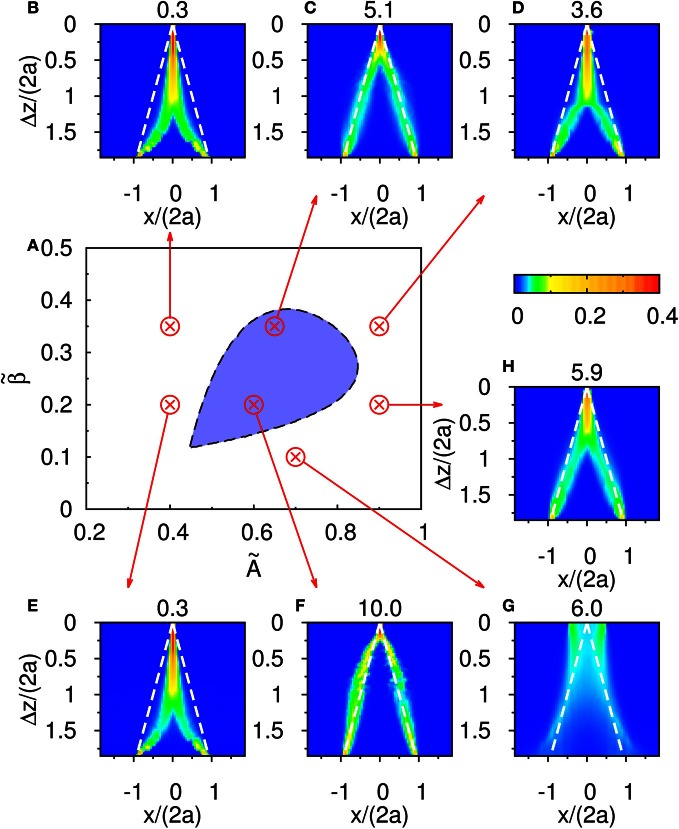
**(A)** The phase diagram of population spikes over the parameter space spanned by ($\tilde{A},\tilde{\beta }$) with the parameter $\tilde{k}=0.5$ and τ_*d*_/τ_*s*_ = 50. **(B–H)** are distributions of the occurence of peak positions as function of Δ*z*. The numbers at the top of **(B–H)** are thresholds used to sample peak positions. Parameters: **(B)** $\tilde{A}=0.4$ and $\tilde{\beta }=0.35$. **(C)** $\tilde{A}=0.65$ and $\tilde{\beta }=0.35$. **(D)** $\tilde{A}=0.9$ and $\tilde{\beta }=0.35$. **(E)** $\tilde{A}=0.4$ and $\tilde{\beta }=0.2$. **(F)** $\tilde{A}=0.6$ and $\tilde{\beta }=0.2$. **(G)** $\tilde{A}=0.7$ and $\tilde{\beta }=0.1$. **(H)** $\tilde{A}=0.9$ and $\tilde{\beta }=0.2$.

The result shown in Figure 4C is not particular for the chosen set of parameters. In Figure 6, there is a phase diagram along with some selected parameters. In Figure 6A, the colored region is the region for population spikes with one stimulus. If $\tilde{A}$ and $\tilde{\beta }$ are chosen from this region, as far as we have observed, similar results can be obtained by choosing appropriate thresholds. If $\tilde{A}$ and $\tilde{\beta }$ are outside the colored region, no matter what the threshold was, the result shown in Figure 4C cannot be reproduced. This result suggests that population spikes are important to resolution enhancement.

### 3.4. Network response with multiple stimuli

**Figure 7:**
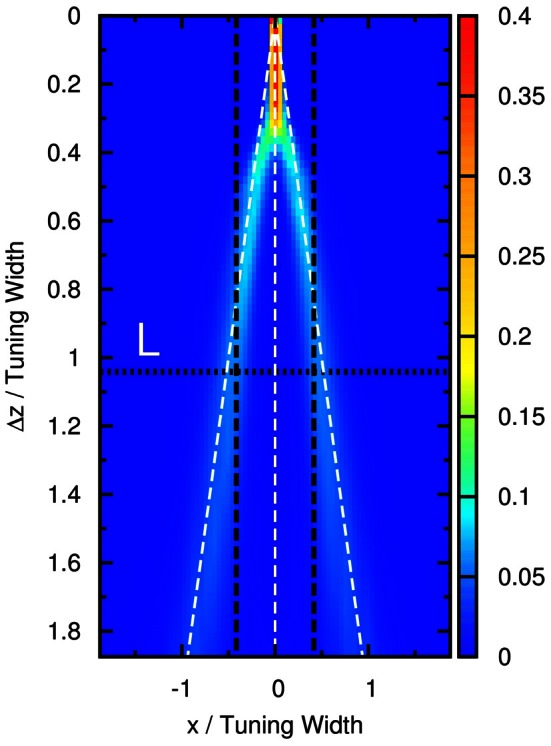
**Contours of the distribution of peak positions higher than 6.2 as a function of preferred stimuli, *x*, and the separation between the two outermost stimuli, Δ*z*, in the case of three equally strong stimuli.** White dashed line: positions of three stimuli. Horizontal dotted line: the case comparable to the three-stimulus experiment reported by Treue et al., 2000. Vertical dashed lines: perception (±40°) reported by subjects in the experiment in units of the tuning width (96°). Parameters: same as Figure 5.

**Figure A2:**
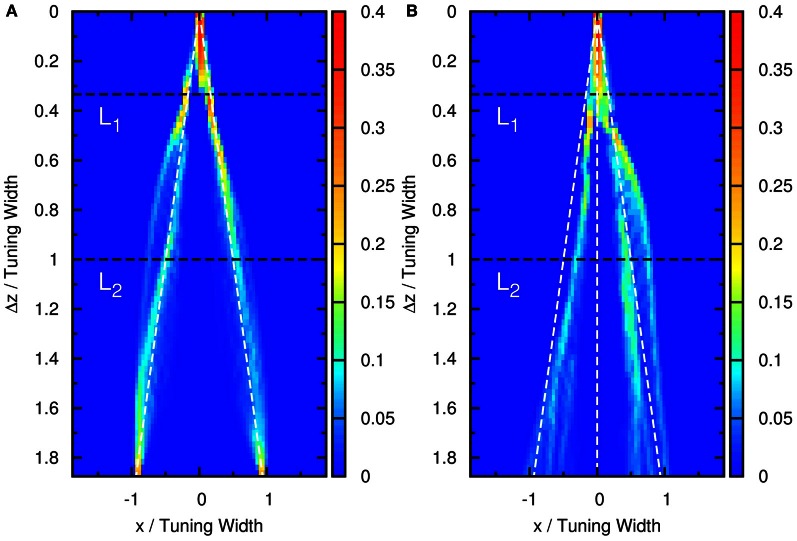
**The population spike occurrence counted within 500τ_*s*_, which is comparable to the timescale in typical experiments. (A)** Situations that there are two stimuli. **(B)** Situations with three stimuli. Other parameters: **(A)** same as Figure 4, **(B)** same as Figure 7.

We further test the response of our model to more than two stimuli. Figure 7 shows the case for three stimuli of equal amplitude, whose peak positions are labeled by the white dashed lines. However, the contours of the distribution of population spikes are double-peaked, similar to those in Figure 5. This result suggests that, if there are three stimuli overlapped together, the network response should give only two groups of neuronal responses. Also, it predicts that peaks of population spikes should occur at positions that underestimate the separation between the outermost stimuli. A similar result for shorter sampling periods comparable to actual experiments can be found in Figure A2B in Appendix.

We found that the experimental result of multiple stimuli reported by Treue et al. is consistent with this prediction. In their paper, it was reported that, when there were three groups of moving dots moving at directions ±50° and 0°, the subjects would report that there were only two moving directions at ±40°. This consistency is shown in Figure 7, where the vertical dotted line *L* labels the position that the outermost stimuli are directed at ±50° when the tuning width is 96°, and the pair of horizontal dashed lines labels ±40° correspondingly.

## 4. Conditions for resolution enhancement

We have demonstrated the phenomenon of resolution enhancement due to modulations of population spikes. To see whether this picture can be generalized to other cases and what alternative models are to be excluded, we summarize the general conditions of its occurrence. To appreciate the significance of each condition, we will consider the alternative scenarios in the presence and absence of the various conditions.

### 4.1. Short-term synaptic depression

**Figure 8:**
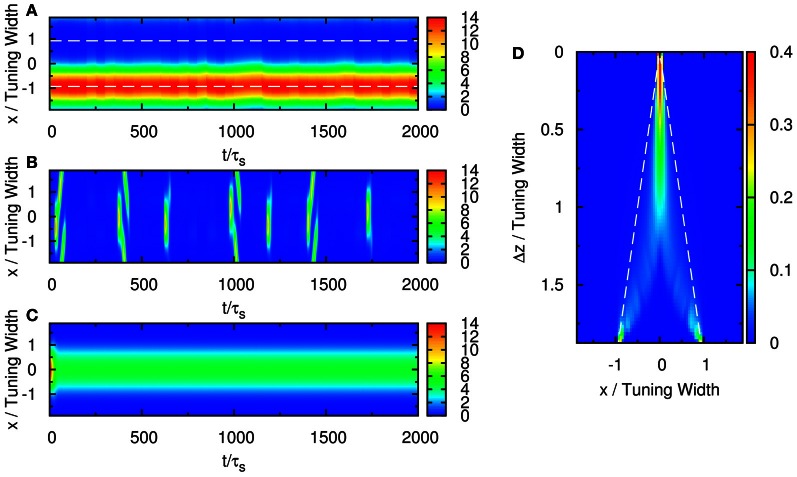
**(A)** Raster plot of firing rate $\tilde{r}$ of the network with two stimuli and without STD. Parameters: $\tilde{k}=0.5$, $\tilde{\beta }=0$, $\tilde{A}=0.8$, *a* = 48π/180, σ_*A*_/*A*_0_ = 0.3, and Δ*z* = 3.1. **(B)** Rastor plot of firing rate $\tilde{r}$ of the network with two stimuli with weak net input profile. Parameters: $\tilde{k}=0.5$, $\tilde{\beta }=0.24$, $\tilde{A}=0.4$, *a* = 48π/180, σ_*A*_/*A*_0_ = 0.3, and Δ*z* = 2.5. **(C)** Rastor plot of firing rate $\tilde{r}$ of the network with two stimuli without height fluctuations in the external input profile. Parameters: $\tilde{k}=0.5$, $\tilde{\beta }=0.24$, $\tilde{A}=0.8$, *a* = 48π/180, σ_*A*_/*A*_0_ = 0, and Δ*z* = 1.67. **(D)** Contours of the distribution of peak positions for all peak heights. White dashed line: positions of the two stimulus components. Parameters: $\tilde{k}=0.5$, $\tilde{\beta }=0.24$, $\tilde{A}=0.8$, *a* = 48π/180, σ_*A*_/*A*_0_ = 0.3, and Δ*z* = 1.0.

Without the STD, the steady state of the neuronal activity profile becomes centered at either one of the two input stimuli. In Figure 8A, when the difference between the input profiles is large, Δ*z*/*a* = 3.7 for instance, the neuronal activity is trapped by the input profile near *x* = 1.55. This case is not consistent with experiments, because when the separation between the input profiles is large enough, the neuronal activity should be able to identify both stimuli. This shows that STD plays the following roles in this phenomenon.

First, STD gives rise to the temporal modulation characterized by the population spikes, in which rapid rises in population activities alternate periodically with drops due to the consumption of neurotransmitters. Spiking activities enable the activity profile to jump from one stimulus position to another easily.

Second, the presence of STD enhances the mobility of the activity profiles. Due to the consumption of neurotransmitters in the active region, the profile tends to relocate itself to less active regions. This is the cause of the increased mobility when the activity profile tracks the movement of external stimuli, as well as their anticipatory tracking as a possible mechanism for delay compensation (Fung et al., 2012a,b). In the parameter regime where the stationary profile becomes unstable in its position, and population spikes become the attractor state, the network tends to establish a population spike in new locations, preventing itself from being trapped by one stimulus. This results in population spikes centered at alternating stimuli and hence the temporal modulation.

**Figure 9:**
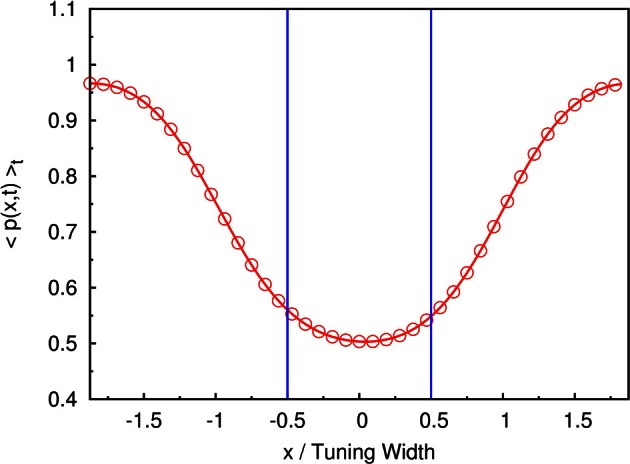
**The time-averaged dynamical variable *p*(*x*, *t*).** Symbols and red line: measurement from the simulation. Blue lines: positions of two stimuli. Parameters: $\tilde{k}=0.5$, $\tilde{\beta }=0.24$, *a* = 48π/180, τ_*d*_/τ_*s*_ = 50, $\tilde{A}=0.8$, and Δ*z* = tuning width of attractor states.

For example, if the two stimuli are strongly overlapped, the average neuronal response concentrates at the in-between region of the two stimuli, as shown in Figure 3B. In this case, the time-average profile of the dynamical variable *p*(*x*, *t*) has a dip centered at the midpoint between two stimuli, as shown in Figure 9. Since, in our model, there are fluctuations of the magnitude of each component of the external input, population spikes occur near the positions of the stimuli, labeled by the blue lines in Figure 9. Since the synaptic efficacies of the presynaptic neurons are stronger in the side region further away from the other stimulus, population spikes are more likely to happen in the outer region rather than the inner region. So, the separation between the two groups of population spikes can be larger than the separation between the two stimuli. This is also the reason why only two groups of population spikes can be observed in the case with three stimuli (Figure 7). STD also explains the slight over-estimation of the perceived positions when the separation of the stimuli is around the tuning width.

**Figure 10:**
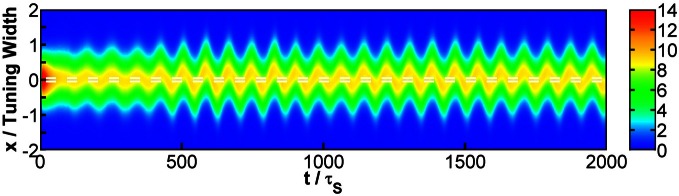
**Raster plot of firing rates $\tilde{r}$ at Δ*z* = 0.1, showing a slosher.** White dashed lines: positions of the stimuli. Other parameters: $\tilde{k}=0.5$, $\tilde{\beta }=0.1$, *a* = 48π/180, $\tilde{A}=0.8$, σ_*A*_/*A*_0_ = 0.2, and τ_*d*_ = 50τ_*s*_.

Third, when STD is not sufficiently strong, we observe that sloshers rather than population spikes are formed (Folias, 2011). These sloshers are bumps that oscillate back and forth around the external stimuli, as shown in Figure 10. The height of the bumps is highest when they slosh to the extreme positions, but due to the weaker STD, the height variation in a cycle is not as extreme as those in the population spikes. The positional extent of their oscillations is mainly determined by the restoring attraction from the external input, and is effectively insensitive to the stimulus profile. Hence in the task of resolving the stimulus directions, the performance is degraded by the very flat part of the curve of the perceived separation when the stimuli have strong overlaps, as shown in Figure 6G.

There are also other variants of the model that demonstrate the significance of STD in similar ways. For example, in recurrent networks with local inhibition, we may replace *B*(*t*) in Equation (3) by *B*′(*x*, *t*) given by

(9) $$B^{\prime }(x,t)=1+\rho k\int dx^{\prime }\exp\;\left(-\frac{{\left|x-x^{\prime }\right|}^{2}}{2b^{2}}\right)u{\left(x^{\prime },t\right)}^{2}.$$

To stabilize the neural activity, the range of the local inhibition, *b*, has to be larger than the range of excitatory connection, *a*. However, if *a* is as large as 48°, this local inhibition can be fairly replaced by *B*(*t*) with appropriate $\tilde{k}$. In the presence of STD, the discrimination performance is comparable to that in Figure 5, but the resolution is poor otherwise.

### 4.2. Suitably strong input profiles

Suitably strong input magnitude is needed to produce the temporally modulated patterns, as illustrated in Figure 6. First, when the magnitude of the external input is too small, no significant system-driven neuronal activity can be observed. Fluctuations of external input components cannot stimulate the population spike, as the activation by input profiles was not strong enough. Second, even when the magnitude of the external input is larger, population spikes can be produced but the stimulus is too weak to pin them at the position of the stimuli. Since the mobility of the population spikes is enhanced by STD, moving population spikes are formed, as illustrated in Figure 8B. Since the population spikes move away from the stimulus positions after their formation, they cannot be used to encode the stimulus positions and also become part of the noisy background affecting the recognition of the stimulus positions. When the stimulus is too strong, population spikes cannot be generated and the resolution degrades.

### 4.3. Fluctuations in input profiles

**Figure 11:**
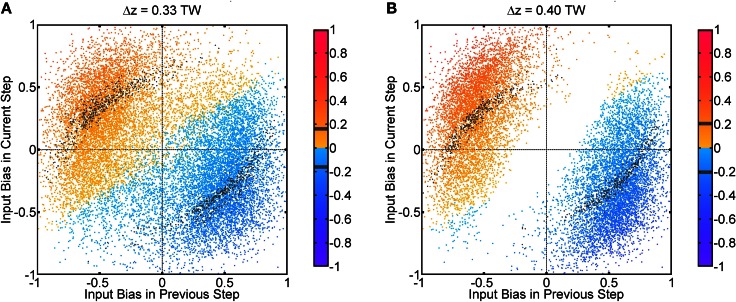
**Population spikes' positions conditional on input fluctuations.** One step refers to 50τ_*s*_, which is the temporal interval between every update in the Gaussian fluctuation δ*A*_*i*_(*t*). Input bias is defined as (δ*A*_1_(*t*) − δ*A*_2_(*t*))/max(*A*_0_ + δ*A*_1_(*t*), *A*_0_ + δ*A*_2_(*t*)). The color code indicates the average position of population spikes above threshold within one step in unit of the tuning width (TW). Gray color means the average position is within the true position of either input ±0.01 TW. True positions of inputs: *z*_1_ = Δ*z*/2, *z*_2_ = − Δ*z*/2. **(A)** Δ*z* = 0.33 TW. **(B)** Δ*z* = 0.40 TW. Parameters: same as Figure 3.

Fluctuations on external input components is important to the behavior in Figure 3. If there were no fluctuations in the input profiles, the net input profile will have only one peak for Δ*z* < 2*a*. As a result, there is effectively one bell-shaped input profile if the difference between two stimuli is too small, and the network response will also be single-peaked, as shown in Figure 8C. Hence fluctuations in the external input play the role of rendering the components distinguishable. As shown in Figure 11, recognition of input location always follows a strong input on the same side at the current step, and a strong input on the other side in the previous step, suggesting that a sudden shift in input bias provides condition for reliable recognition. In fact, the noise fluctuations act as the signals themselves, without which the single-peaked input provides little information about the components. Results in Figure 11 also illustrate that, statistically, the system is able to give valid responses to stimulus changes in a single step. This explains why the network yields discrimination performance equally well for short and long sampling periods, as demonstrated in a comparison between Figures 4C, A2A.

The fluctuations may come from randomness in the inputs. Psychophysical experiments show that spatial and temporal randomness is important for perceptions of motion transparency. For example, regularly spaced lines moving in opposite directions do not give the perception of transparent motion, whereas randomly spaced lines are able to do so (Qian et al., 1994). The input signals come from different locations of the visual field, and fluctuations arise when the perceived objects move from one location to another. Fluctuations may also arise when feedback signals from advanced stages of processing guide the system to shift its attention from one specific component to another.

Functionally, fluctuations facilitate the resolution of the directional inputs in the following two aspects. Spatially, it breaks the symmetry of the input profile. Temporally, it provides the time-dependent signals that induce the population spikes centered at the component that happens to be strengthened by fluctuations. This enables the system to recognize the temporally modulated inputs. On the other hand, for systems processing only time-averaged inputs, the height fluctuations vanish when averaged over time, so that the components cannot be detected.

### 4.4. Thresholding

Even after temporal modulation, resolution based on the network response can still carry large errors. As shown in Figure 3C, there are obviously two groups centering around the positions of the two components, but in between the two components, there is a region with moderate neuronal activities. If the network includes neuronal activities of all magnitudes, the errors in estimating the component positions will be large, especially when Δ*z* is small. Indeed, Figure 8D shows that without imposing any thresholds on the neuronal activities, the network cannot resolve the two components until the separation exceeds the tuning width.

**Figure 12:**
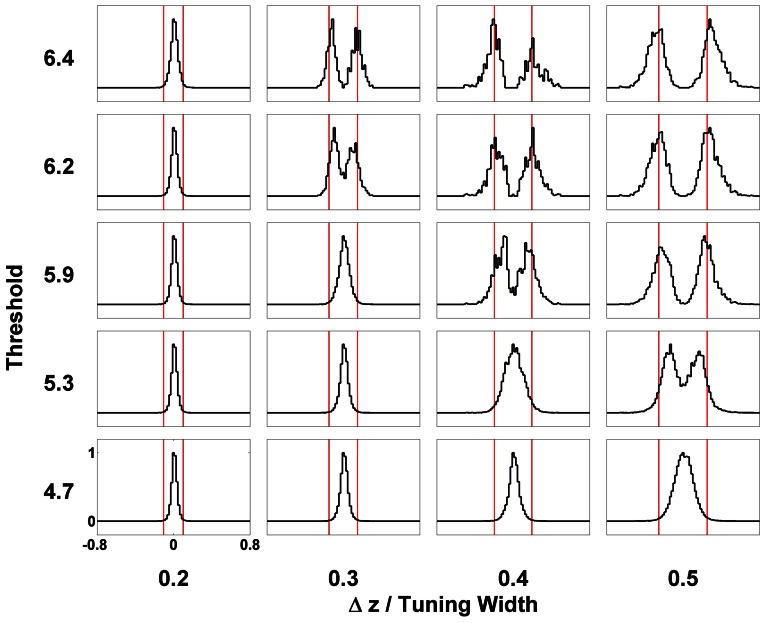
**Effect of thresholding on the statistics of peak positions.** Distributions of peak positions higher than different thresholds (shown on the left) when two stimuli are separated by Δ*z* (shown at the bottom) are plotted in patches. The scales of all the patches are the same, shown on the lower-left patch. Distributions are normalized to the maximum value. Red bars mark the positions of two stimuli. Other parameters: $\tilde{k}=0.5$, $\tilde{\beta }=0.1$, *a* = 48π/180, $\tilde{A}=0.8$, σ_*A*_/*A*_0_ = 0.2, and τ_*d*_ = 50τ_*s*_.

In order to solve this problem, we introduce a threshold on the maximum firing rates. We collect statistics of the peak positions of the firing rate profile when their height exceeds the threshold. The result is shown in Figure 5, indicating a significant improvement of resolution compared with Figure 8D. The effects of the threshold value on the resolution performance are shown in Figure 12. When the threshold is low, the components are not resolved even at a separation of 0.5 times the tuning width. On the other hand, when the threshold is too high, the statistics of peak positions becomes too sparse to be reliable. In an intermediate range of thresholds that is not too narrow, the resolution of the components can be achieved down to separations of 0.3–0.4 times the tuning width.

### 4.5. Recurrent connections

Finally, we would like to stress the importance of recurrent connections in achieving resolution enhancement. With no recurrence, population spikes cannot be generated and the amplification of the difference between nearly overlapping inputs cannot be achieved. Let us consider a purely feedforward network, with weaker but spatially broader inhibition than excitation,

(10) $$\tau_{s}\frac{du}{dt}(x,t)=-u(x,t)+\rho \int dx^{\prime }[J_{E}\exp\left(-\frac{{\left|x-x^{\prime }\right|}^{2}}{2a^{2}}\right)-J_{I}\exp\left(-\frac{{\left|x-x^{\prime }\right|}^{2}}{2b^{2}}\right)]p\left(x^{\prime },t\right)I^{\text{ext}}\left(x^{\prime },t\right)$$

(11) $$\tau_{d}\frac{dp}{dt}(x,t)=-p(x,t)+1-\tau_{d}\beta p(x,t)I^{\text{ext}}(x,t)$$

**Figure 13:**
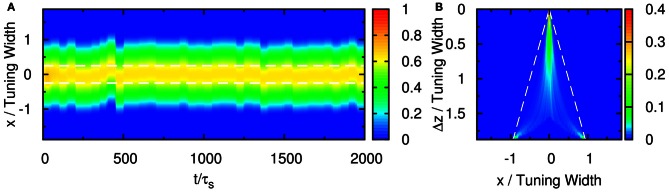
**(A)** Raster plot of firing rate *r* for purely feedforward network. Δ*z* = 0.5 TW. White dashed line: positions of the two stimuli. **(B)** Contours of the distribution of peak positions higher than 0.45 as a function of preferred stimuli,*x*, and the separation between the two stimuli, Δ*z*. White dashed line: positions of the two stimuli. Parameters: *J*_*I*_ = 0.3*J*_*E*_, *b* = 3*a*, τ_*d*_β/ρ*J*_*E*_ = 0.2, ρ*J*_*E*_*A* = 0.8, σ_δ*A*_*i*__/*A*_0_ = 0.3, and τ_*d*_ = 50τ_*s*_.

(12)        r(x,t)=Θ[u(x,t)]u(x,t),

where *J*_*E*_ > *J*_*I*_ and *I*^ext^(*x*, *t*) is the same as that in recurrent network in Equation (6). Although in this feedforward network STD can still modulate the synaptic efficacy so that neuronal activities prefer the side region to the midpoint between two stimuli, temporal modulation, which is essential to population spikes, cannot be realized without feedback. As mentioned above, population spikes make it easier for the activity profile to switch off on one side and grow up on the other. As shown in Figure 13, the resolution enhancement in the purely feedforward network is poor. In fact, the behavior is very similar to those in the non-spiking region even when the architecture is recurrent, as shown in Figures 6A,E.

## 5. Discussion

In this paper, we have demonstrated how STD plays the role of generating population spikes that can carry information extra to spike rates. We have used the example of resolving transparent motion with two components in a continuous attractor neural network, and have shown that the temporal modulation of the firing rates enables the network to enhance the resolution of motion transparency, thereby providing a possible explanation to the longstanding mystery of resolving separations narrower than the tuning width of the neurons, and resulting in input-output relations that can have excellent agreement with experimental results (Treue et al., 2000). The role played by STD was further clarified by comparison with alternate scenarios under 4 general conditions.

First, the strength of STD should be sufficiently strong. Weaker STD may result in the network response being pinned by one of the two components, or slosher modes that span a range of positions effectively independent of the component separations. On the other hand, sufficiently strong STD can give rise to population spikes, endowing them the freedom to alternate between the two components. Equally important is the provision of temporal modulation by the population spikes, so that the firing patterns indeed contain information of the stimuli, even though the time-averaged firing rate can only resolve separations larger than the tuning width of neurons, as shown in Figure 4 and found experimentally by Treue et al. (2000). The role played by temporally modulated signals in transparent motions can be tested in future experiments.

Second, the strength of the input should be sufficiently strong. Otherwise, no population spikes can be produced. Even for moderately strong input, the population spikes become moving ones, and fail to represent the stimulus positions.

Third, fluctuations in the input profiles are also important. They provide the temporally sensitive signals when the two components cannot be resolved in the time-averaged input. They correspond to the “unbalanced motion signals” in the detection of transparent motion with opposite moving directions (Qian et al., 1994).

Fourth, thresholds are needed to extract the information of the stimuli contained in the firing patterns, since they are able to truncate background activities that interfere the signals from the two components.

Our proposed model is not the first model or mechanism to explain the behavior of the discriminational task in transparent motion experiments. It was suggested that the curvature of the average neural activity may provide information of multiple stimuli, but the neural activity is wider than expected (Treue et al., 2000). Other proposals require more complex structures to achieve the task. For example, a population to encode uncertainty is needed to differentiate between multiplicity and uncertainty (Sahani and Dayan, 2003), and additional internal structures are needed to provide feedback information (Raudies et al., 2011). While admittedly involving additional structures and layers can augment the functionality of the brain, our work shows that it is possible to achieve with little additional structure the performance consistent with experiments in Treue et al. (2000) and Braddick et al. (2002). An interesting future direction is to consider whether firing rates multiplexed with temporal modulations can be an instrument to achieve the differentiation between multiplicity and uncertainty posed in Sahani and Dayan (2003).

The ability of STD to generate temporally modulated response is also applicable to other brain tasks, such as switching between percepts in competitive neural networks (Kilpatrick, 2012). Compared with other conventional neural network models processing time-averaged or static neuronal response profiles, the temporal component provides an extra dimension to encode acute stimuli, so that information processing performance can be significantly enhanced.

### Conflict of interest statement

The authors declare that the research was conducted in the absence of any commercial or financial relationships that could be construed as a potential conflict of interest.
